# Factors influencing the speed of cancer diagnosis in rural Western Australia: a General Practice perspective

**DOI:** 10.1186/1471-2296-8-27

**Published:** 2007-05-04

**Authors:** Moyez Jiwa, Georgia Halkett, Samar Aoun, Hayley Arnet, Marthe Smith, Megan Pilkington, Cheryl McMullen

**Affiliations:** 1 Western Australian Centre for Cancer and Palliative Care (WACCPC), Curtin University of Technology, Perth, Western Australia; 2 Western Australian Centre for Cancer and Palliative Care (WACCPC), School of Nursing, Midwifery and Postgraduate Medicine, Edith Cowan University, Perth, Western Australia; 3 University of Western Australia, 35 Stirling Highway, Crawley 6009, Western Australia

## Abstract

**Introduction:**

The speed of diagnosis impacts on prognosis and survival in all types of cancer. In most cases survival and prognosis are significantly worse in rural and remote Australian populations who have less access to diagnostic and therapeutic services than metropolitan communities in this country. Research suggests that in general delays in diagnosis were a factor of misdiagnosis, the confounding effect of existing conditions and delayed or misleading investigation of symptoms. The aim of this study is to further explore the factors that impact on the speed of diagnosis in rural Western Australia with direct reference to General Practitioners (GPs) working in this setting.

**Methods:**

The methodology consisted of a structured discussion of specific cases. GPs based in two rural locations in Western Australia were asked to identify up to eight clinical cases for discussion. A diversity of cases was requested encompassing those with timely and delayed diagnosis of cancer. Focus groups were held with the practitioners to identify which factors under six headings delayed or facilitated the diagnosis in each case. A structured summary of the discussion was relayed to a wider group of GPs to seek additional views or comments on specific factors that impact on the speed of cancer diagnosis in rural and remote locations in Australia.

**Results:**

A number of factors affecting the speed of diagnosis were identified: the demographic shift towards a frailer and older population, presenting with multiple and complex diseases, increases the challenge to identify early cancer symptoms; seasonal and demanding work patterns leading to procrastination in presenting for medical care; unhelpful scheduling of specialist appointments; and the varying impact of informal networks and social relationships.

**Conclusion:**

Within the limitations of this study we have generated a number of hypotheses that require formal evaluation: (1) GPs working within informal professional and social networks are better informed about their patients' health needs and have an advantage in making early diagnosis; (2) Despite the other differences in the population characteristics decentralising services would improve the prospect for timely diagnosis; and (3) Careful coordination of specialist appointments would improve the speed of diagnosis for rural patients.

## Background

Cancer is the leading cause of death in Australia; the annual incidence rate exceeds 88,000 cases resulting in over 36,000 mortalities each year. One in three Australian men and one in four Australian women will be affected by cancer before the age of 75 [1]. Early diagnosis is critical in achieving a better prognosis in all types of cancers. The literature suggests that patients who live in rural locations have relatively poorer outcomes for many but not all chronic and malignant conditions although this is not apparent for all diseases. An Australian team from New South Wales demonstrated that differences in all-cause mortality between rural and metropolitan populations were magnified by 2–3% every 5 years [2]. Similarly, figures from South Australia demonstrated that, even though the overall five year survival for all cancers combined are similar in rural and metropolitan areas, there were some differences in incidence rates for 11 of the 31 types of cancer. In ten of these types of cancer survival was better in metropolitan as opposed to rural populations [3]. Echoing these findings, researchers in Queensland found that the adjusted case mortality rates for cutaneous melanoma was 20% higher in rural areas and a variety of factors including early detection impacted on survival [4].

Cancer services in Australia are principally located in capital cities. However 30% of the population live in rural and remote areas, therefore it is postulated that access to services in remote areas may have a bearing on poorer outcomes [5]. There is some evidence that specialist outreach visits to remote disadvantaged communities in Australia improves access to the requisite procedures and is cost effective [6]. Indeed delays in lung cancer diagnosis are reported elsewhere to be a function of longer waiting times for investigation of symptoms [7]. It is also suggested that differences in health beliefs in rural areas might exert a significant influence on health seeking behaviour [8-10]. Such findings are repeated internationally as in a survey of patients the UK where it was demonstrated that delayed diagnosis of colorectal cancer in rural areas was a function of: late presentation; inadequate arrangements for investigation; and communication failures [11,12]. Bain et al further concluded that continuity of care in rural locations sometimes contributed to delays when the primary care doctor was "locked into the wrong diagnosis" and the patient was unable to seek a second opinion. This was echoed in a recent systematic review in relation to upper gastrointestinal cancers where the main factors related to practitioner delay were reported to be misdiagnosis, application and interpretation of tests, and the confounding effect of existing disease [13]. Rural patients were also found to have lower expectations and in general faced more hurdles in their trajectory through the health care system. Therefore demographic, socio-economic, environmental and other rural-specific factors all seem to contribute to inequity and poorer health status in rural areas [12].

As far as we are aware no exploratory data have been reported on the speed of cancer diagnosis with reference to General Practitioners (GPs) in rural Western Australia. The aim of this study is to explore the factors that impact on the speed of diagnosis in this location prompted by the experience of practitioners at the frontline in the diagnosis of cancer.

## Methods

### Ethics

Ethics approval was granted by the HREC at the University of Western Australia (RA/4/1/1461).

### Setting

The study was conducted in two rural locations in Western Australia: Geraldton, a relatively large city located approximately 403 km north of Perth (the capital of Western Australia); and Manjimup, a smaller town located 306 km south of Perth. These locations were chosen to accommodate the needs of the medical student researchers (MP & CMcM) participating in this project. Features of these locations including the incidence of cancer and demographic details are summarised in Table 1.

**Table 1:** Features of selected locations for study [23-26]

Location	Population	Median Age	Population over 65 yrs	Main Industries	Incidence of cancer 1998–2002
Geraldton and surrounding areas	32,635 [27]	36 yrs	10% [28]	Agriculture, fishing, tourism centred on beach	520 [29]
Manjimup and surrounding areas	10,030 [30]	37 yrs	11% [31]	Forestry, agriculture [28]	174 [32]

### Methodology

The methodology followed two steps:

1. Focus group discussions with GPs about specific cases from their practices regarding factors that delayed or facilitated the diagnosis of cancer. Verbal consent was obtained for participation in the focus group.

2. Postal consultation of a wider group of practitioners, in tabular format, seeking consensus about the findings of Step 1. Participation in the postal survey was taken as consent.

#### Step 1

GPs located in Geraldton and Manjimup involved in undergraduate medical training and those who were identified to the team by such practitioners were invited to participate in a focus group discussion (n = 42). In this first stage, participating practitioners were asked to identify up to eight cancer cases each as a focus for the subsequent discussions.

The participants were asked to submit cases with one or more of these criteria:

1. Early diagnosis

2. Late presentation

3. Diagnosis following screening test (e.g. mammography)

4. Presenting with atypical symptoms or features and

5. Delayed diagnosis for some other reason.

These criteria were selected to increase to reflect the range of pathways and stages of disease for patients entering specialist services. The following information was requested for each case:

a) Date of presentation with symptoms and signs referable to the relevant system or that suggested the need for investigation;

b) Whether GPs made a routine or urgent referral;

c) Interval from presentation to diagnosis and

d) Interval from diagnosis to definitive treatment for the relevant cancer, where appropriate.

**Figure 1:**
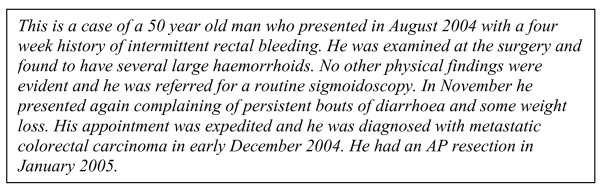
Case of colorectal cancer as presented in focus group discussions (Step 1).

After case summaries were submitted by the participants the researchers (CM/MP) selected six cases for discussion in the focus groups. The most common cancer types presenting in Australian practice [14,15] were selected; prostate cancer, breast cancer, colorectal cancer, bronchus/lung cancer and melanoma. All patient identifiers were removed to protect patient privacy. Typically cases were presented as per Figure 1.

In both locations, a structured review of clinical incidents, akin to a case conference, was performed according to the protocol outlined by the Clinical Risk Unit [16]. The approach includes a systematic exploration of the issues through case analysis. The methodology developed for use in analysing industrial accidents has been adapted for use in a healthcare setting, classifying 'error producing circumstances' and 'organisational factors' in a single broad framework of factors affecting clinical practice [16]. This method was previously used in a similar study of UK general practitioners and involves inviting comments about the cause of delayed diagnosis and summarising the discussion on a white board and recording comments verbatim [17]. During discussion, the key factors influencing the speed of cancer diagnosis for individual cases were examined under the following six headings, and participants were asked to focus on factors that they considered of particular importance in the context of rural clinical practice:

 Patient characteristics (employment or attitudes to health care)

 GP/Specialist factors (Characteristics of health care provider)

 Task factors (protocols for establishing a diagnosis)

 Team factors (e.g. communication)

 Work environment (e.g. available resources or constraints)

 Organisational management and institutional factors (e.g. access to local clinics).

#### Step 2

After the focus group discussion the GPs' views were summarised as in Table 2, with an additional column for comments. The table categorised the identified factors impacting on diagnosis under the headings adopted in Step 1. This table was subsequently circulated to all practitioners previously invited to participate in Step 1 for further comment and to seek endorsement for the views expressed in the focus group.

**Table 2:** Factors influencing the speed of cancer diagnosis

Factor	Delaying diagnosis	Facilitating diagnosis
Patient	Need to travel to clinics in the capital may have financial and logistical implications for the patient and therefore lead to procrastination.	Patients in rural areas more likely to comply with GPs advice or attend appointments.
Health professional	Different gender of GP may deter **some **patients from presenting with embarrassing symptoms that require intimate examination for diagnosis.	Greater continuity of care. Quality of doctor-patient relationship
Task	Equivocal tests necessitate repeat visits to clinic. Inaccessible guidelines.	Some conditions can be managed by local GPs or by availability of local facilities.
Team	Lack of coordination for individual patients' needs may result in inconvenient scheduling of appointments. Limited scope to obtain second opinions.	Excellent communication and local professional networks.
Work Environment	Short consultations for multiple and undifferentiated medical complaints especially in older patients.	More comprehensive knowledge about the patient and the social context may be helpful in clinical assessment.
Organisational	Access to specialists limited by distance from state capital.	Visiting specialists may reduce burden of travel for patient.

## Results

One 'focus group' was held in each location. Overall, Twenty five percent of the invited GPs participated in the focus group component of the study (Step 1) and 100% responded to the summary of the group's conclusions in Step 2. Those who participated in the focus group were a self selecting/interested practitioners. In both locations, a number of factors were identified that can either delay or facilitate diagnosis of cancer in rural and remote locations. The key findings in relation to rural practice summarised in (Table 2) were endorsed by all participating GPs and includes new information that was forthcoming after the focus group.

The key themes highlighted in Table 2 and the comments recorded verbatim are further expanded under the following headings: limited access to specialists and GPs; the diagnostic and consultation process; early cancer symptoms and social networks.

### Limited access to specialists and GPs

One of the main barriers to speedy diagnosis of cancer in rural and remote areas is limited access to health services. Many diagnostic and therapeutic services are centralised to the state capital, Perth, several hundred kilometres from either location. Attending appointments so far from home implies loss of income as well as travel and accommodation expenses for the patient. For patients in blue collar occupations and for those with seasonal businesses in the area, such expense may be unacceptable and procrastination may lead to significant delays in diagnosis. The tyranny of distance is exacerbated when diagnostic tests need to be repeated and patients face further loss of earnings. Therefore, people in rural areas may choose to wait until the specialist makes a visit to the country area rather than travel to the nearest regional or capital city. Help is limited insofar as eligibility for government assistance with travel based on narrow criteria and the scheme is thought to be bureaucratic and inflexible. Participating GPs suggested that delays can also be attributed to a lack of adequate coordination of patient appointments. The result is a succession of different appointments focusing on different aspects of the patient's condition. A need to organise repeated trips to the metropolitan centre was reported to be the reason why some patients choose to delay seeking help for significant symptoms. Aligned to this limited access to specialists, is the prolonged waiting time for appointments with GPs in rural communities. The publicised shortage of GPs in rural and remote regions implies that patients may have to wait a number of weeks to see their GP of choice. This factor significantly increases the prospect of delayed presentation for those who only choose to disclose symptoms to a GP with whom they are comfortable or familiar.

### The diagnostic and consultation process

Referral guidelines are updated frequently in Australia to the extent that GPs felt "swamped by literature". It was suggested that the guidelines and protocols for referral need to be simplified and more readily accessible for them to function effectively. In practice, GPs may respond by completing as much of the diagnostic work-up as possible to minimise inconvenience for the patient. In light of the potential disruption and inconvenience to those referred, GPs may also be more selective about which cases are referred and choose to refer later in the diagnostic process. In rural communities there is also a tendency for patients to present with multiple issues at consultation. With the demise of the timber industry and increasing number of older people moving to the area to retire at one study site, there has been a demographic shift away from young, relatively healthy workers, to a population of older people with multiple and complex medical problems. The habit of "saving up" health concerns until a convenient occasion to visit town, perhaps combined with other business (e.g. shopping) leads to further pressure to remain vigilant to "red flag" cancer symptoms in the consultation. As one of the GPs stated, "More is missed by not looking than not knowing". Within the older population, cognitive impairment and social isolation may contribute to delayed presentation because symptoms may go unnoticed. Speed of diagnosis was also hindered by people refusing to accept the "sick role"; the stereotype that "the strong farmer doesn't get ill" was reported to have a grain of truth.

### Early cancer symptoms

It was thought that patients "did not know what was important and what was not important" in relation to specific symptoms. There was a tendency to disregard "minor" symptoms if they were weighed up against the inconvenience of making an appointment. On the one hand, GPs thought that patients are becoming better informed, largely due to the advent of the internet. On the other hand, this was not held to be true of older patients and it is this population that is at highest risk of cancer [15,18]. Notably, GPs reported that skin cancers were the most frequently diagnosed cancer due in part to the outdoor lifestyle of rural people. In such cases, the system of referral and diagnosis was perceived as less important because GPs tend to treat these cases themselves.

### Social networks

Close social networks between patients and their health carers in rural communities could be both a help and a hindrance to cancer diagnosis. For example, the "small town relationship" can assist some patients in obtaining appointments or disclosing symptoms earlier. However, social interaction between GP and patient in a small town sometimes means that patients are reluctant to disclose "embarrassing" symptoms e.g. diarrhoea or a breast lump, especially to a GP of the opposite sex with whom they or their families may have a social relationship. In the past there was also a potential for communication difficulties between GP and patient due to the large number of migrant workers, many of whom were non-English speakers (e.g. seasonal fruit pickers at one site). There was also a potential cultural barrier with Indigenous patients, who tended to present late and at a later stage of disease than non-Indigenous patients.

## Discussion

Given that 30% of the population of Australia live in rural and remote locations three findings in this study were of particular interest; first that the demographic shift towards an older population and the seasonal and/or demanding work patterns in rural Australian communities have profound implications for timely access to healthcare; second, that coordination of clinical appointments is of particular importance in rural communities and finally, that informal networks and social relationships in these communities have a significant bearing on speed of diagnosis.

GPs in this study highlighted the difficulties of dealing with patients who 'save up' presenting symptoms until they have other reasons to come to town. This reflects what we already know about the differences in expectation and health seeking behaviour of rural people as reviewed in the background to this study. Of particular note however is the underestimated consequence of ageing as it impacts on the consultation. Older patients are more likely to present multiple and sometimes mutually exclusive medical problems as a consequence of ageing. It has also been demonstrated previously that patients in rural areas consult less often and present more clinical issues than equivalent metropolitan patients further increasing the complexity of the consultation [19,20]. The potential impact may be gleaned from non-medical research which proposes that 'multi-tasking' can lead to critical errors [21]. The Australian 'BEACH' study reviewed consultations in which malignancies were diagnosed and reported the reasons for the encounter [14]. A significant number of consultations were coded with multiple ICPC-2 (International Classification of Primary Care) codes, indicating that practitioners were asked to address other problems during consultations in which cancer symptoms were also presented. The extent to which this may have delayed the diagnosis of the relevant cancer is unknown and may be the confounding effect of existing conditions reported by MacDonald et al as a cause of delayed diagnosis [13]. In this context the implementation of referral guidelines remains problematic. Practitioners expressed the view that compliance with guidelines was tempered by the inconvenience occasioned to the patient when referred to clinics a long distance from home. Previous Australian surveys have reported poor GP compliance with guidelines [22]. It has been emphasised that guidelines should be more effectively disseminated. Our data suggests that guidelines also need to take account of the context of rural practice.

The role of geographical isolation emphasised by participants here was previously reported in Western Australia by Rankin et al. [23] as a key factor in delay in accessing specialist medical services. It was estimated in 2001 that a saving of AU$1077 was made per specialist consultation when accessing a local rather than a metropolitan service. Savings were observed in travel time, distance travelled, lost income, provision of an escort and waiting time. The positive value of outreach clinics to Australians in rural and remote regions was also reported by Gruen and colleagues in the *Lancet *[6]. Although one might argue that the provision of outreach clinics to all rural and remote communities is unrealistic, clinicians in the current study suggested that there is scope to refine the help that is already available and to coordinate a more efficient service. However, factors that influence cancer survival, and possibly treatment, may reach beyond remoteness of residence and ability to pay. Again, as emphasised by the GPs, and in the literature, they include knowledge, attitudes and beliefs about cancer which may influence presentation of symptoms and completion of treatment [24]. With regard to 'red flag' cancer symptoms further delays are possible if patients have to be redirected in secondary care. For many patients without private insurance it might mean having to enter waiting lists in another specialty and further delay.

Participants emphasised the importance of social networks in the diagnostic process in their practice. The role of such networks in cancer has been acknowledged by a team from Queensland, who reported that the rural patient's network of family, friends and community can, and does, play an active role in the provision of emotional and practical support for the cancer patient [25]. However, others have also concluded that social relationships play an important role in health and help seeking behaviour generally [26]. The importance of this finding relates to interventions aimed at improving early recognition, presentation and survival from cancer in rural communities. The findings of the current study suggest that these informal networks and relationships may significantly improve the prospects for early diagnosis in cancers that rely on early recognition of symptoms. Further research needs to be conducted to determine what steps can be taken to improve the speed of diagnosis in rural locations.

### Limitations

The views reported in this study are taken only from the perspective of GPs at two locations that were convenient for the team. We cannot confirm that these practitioners were necessarily representative of colleagues elsewhere in Western Australia. Initial discussions with GPs were based on actual clinical experience and prompted by details from cases identified by practitioners themselves. It was possible that the selection of such cases may have been biased towards cases with a more favourable trajectory. However, the range of issues raised in the wider discussion suggested that the impact of this was minimal and that an extensive range of complex issues were identified scanning the full patient journey from presentation to diagnosis. To gain a broader perspective of the issues involved future research needs to include patients' perspective and the perspectives of the specialists and other service providers involved.

## Conclusion

Although findings cannot be generalised to all rural areas, a number of untested hypotheses have been generated. These hypotheses need to be formally evaluated and additional research needs to be conducted to improve the speed of diagnosis in rural and remote Australia: (1) GPs working within informal professional and social networks are better informed about their patients' health needs and have an advantage in making early diagnosis; (2) Despite the other differences in the population characteristics decentralising services would improve the prospect for timely diagnosis; and (3) Careful coordination of specialist appointments would improve the speed of diagnosis for rural patients.

## Competing interests

The author(s) declare that they have no competing interests.

## Authors' contributions

All authors contributed equally to this study.

## Pre-publication history

The pre-publication history for this paper can be accessed here:



## References

[B1] AIHW, AACR (2004). Cancer in Australia 2001. Cancer Series No 28.

[B2] Hayes LJ, Quine S, Taylor R (2005). New South Wales trends in mortality differentials between small rural and urban communities over a 25-year period, 1970–1994. Aust J Rural Health.

[B3] Wilkinson D, Cameron K (2004). Cancer and Cancer Risk in South Australia: What Evidence for a Rural-Urban Health Differential?. Aust J Rural Health.

[B4] Coory M, Smithers M, Baade P, Aitken J, Ring I (2006). Urban-rural differences in survival from cutaneous melanoma in Queensland [Cancer prevention and control]. Aust N Z J Public Health.

[B5] AIHW (1998). Health in rural and remote Australia.

[B6] Gruen RL, Bailie RS, Wang Z, Heard S, O'Rourke IC (2006). Specialist outreach to isolated and disadvantaged communities: a population-based study. Lancet.

[B7] Bjerager M, Palshof T, Dahl R, Vedsted P, Olesen F (2006). Delay in diagnosis of lung cancer in general practice. Br J Gen Pract.

[B8] Aoun S, Donovan RJ, Johnson L, Egger G (2002). Preventive Care in the Context of Men's Health. J Health Psychol.

[B9] Aoun S, Johnson L (2002). Men's Health Promotion by General Practitioners in a workplace setting. Aust J Rural Health.

[B10] Howat A, Veitch C, Cairns W (2006). A descriptive study comparing health attitudes of urban and rural oncology patients. Rural Remote Health.

[B11] Bain NSC, Campbell NC (2000). Treating patients with colorectal cancer in rural and urban areas: a qualitative study of the patients' perspective. Fam Pract.

[B12] Bain NSC, Campbell NC, Ritchie LD, Cassidy J (2002). Striking the right balance in colorectal cancer care – a qualitative study of rural and urban patients. Fam Pract.

[B13] Macdonald S, Macleod U, Campbell NC, Weller D, Mitchell E (2006). Systematic review of factors influencing patient and practitioner delay in diagnosis of upper gastrointestinal cancer. The Br J of Cancer.

[B14] Charles J, Harrison C, Britt H (2006). Malignant neoplasms – Management in Australian General Practice. Aust Fam Physician.

[B15] McDermoid I, AIHW, AACR, NCSG (2005). Cancer incidence projections, Australia 2002 to 2011.

[B16] Clinical Risk Unit. http://www.patientsafety.ucl.ac.uk/caseanalysis.htm.

[B17] Jiwa M, Reid J, Handley C, Grimwood J, Ward S, Turner K, Ibbotson M, Thorman N (2004). Less Haste More Speed: factors that prolong the interval from presentation to diagnosis in some cancers. Fam Pract.

[B18] Beechy-Newman N, Kulkarni D (2004). Cancer in older patients. Rev Clin Gerontol.

[B19] Knox S, Britt H (2004). The contribution of demographic and morbidity factors to self-reported visit frequency of patients: a cross-sectional study of general practice patients in Australia. BMC Fam Pract.

[B20] Britt HC, Valenti L, Miller GC Determinants of consultation length in Australian general practice. Med J Aust.

[B21] Lien M-C, McCann RS, Ruthruff E, Proctor RW (2005). Dual-Task Performance With Ideomotor-Compatible Tasks: Is the Central Processing Bottleneck Intact, Bypassed, or Shifted in Locus?. J Exp Psychol Hum Percept Perform.

[B22] Sladden MJ, Thomson AN (1998). How do general practitioners manage rectal bleeding?. Aust Fam Physician.

[B23] Rankin SL, Hughes-Anderson W, House AK, Heath DI, Aitken RJ, House J (2001). Costs of accessing surgical specialists by rural and remote residents. Aust N Z J Surg.

[B24] Jong KE, Vale PJ, Armstrong BK (2005). Rural inequalities in cancer care and outcome. Med J Aust.

[B25] McGrath P, Patterson C, Yates P, Treloar S, Oldenburg B, Loos C (1999). A study of postdiagnosis breast cancer concerns for women living in rural and remote Queensland. Part II: Support Issues. Aust J Rural Health.

[B26] Calnan M (1983). Social networks and patterns of help-seeking behaviour. Soc Sci Med.

[B27] MWDC Population. http://www.mwdc.wa.gov.au/default.asp?documentid=18.

[B28] A snapshot of Geraldton. http://www.abs.gov.au/Ausstats/ABS@cpp.nsf/o/04f0487B920595CDCA25710A0017EF70?Open.

[B29] Threlfall T, Powers K, Langley J (2004). Cancer in Western Australia, 1998–2002: incidence and mortality by statistical local area (SLA). Statistical Series No 72.

[B30] Shire of Manjimup. http://www.councils.wa.gov.au/directory/council_websites/manjimup/.

[B31] A snapshot of Manjimup. http://www.abs.gov.au/Ausstats/ABS@cpp.nsf/o/0106c19241A6B366CA25710A001787Ec?open.

[B32] Shire of Manjimup, Demographics Overview. http://www.manjimup.wa.gov.au.

